# Examining the Regional Co‐Variability of the Atmospheric Water and Energy Imbalances in Different Model Configurations—Linking Clouds and Circulation

**DOI:** 10.1029/2021MS002951

**Published:** 2022-06-04

**Authors:** Guy Dagan, Philip Stier, Beth Dingley, Andrew I. L. Williams

**Affiliations:** ^1^ Fredy and Nadine Herrmann Institute of Earth Sciences Hebrew University Jerusalem Israel; ^2^ Department of Physics, Atmospheric, Oceanic and Planetary Physics University of Oxford Oxford UK

**Keywords:** clouds, water budget, energy budget, circulation, tropics, climate change

## Abstract

Clouds are a key player in the global climate system, affecting the atmospheric water and energy budgets, and they are strongly coupled to the large‐scale atmospheric circulation. Here, we examine the co‐variability of the atmospheric energy and water budget imbalances in three different global model configurations–radiative‐convective equilibrium, aqua‐planet, and global simulations with land. The gradual increase in the level of complexity of the model configuration enables an investigation of the effects of rotation, meridional temperature gradient, land‐sea contrast, and seasonal cycle on the co‐variability of the water and energy imbalances. We demonstrate how this co‐variability is linked to both the large‐scale tropical atmospheric circulation and to cloud properties. Hence, we propose a co‐variability‐based framework that connects cloud properties to the large‐scale tropical circulation and climate system and is directly linked to the top‐down constrains on the system—the water and energy budgets. In addition, we examine how the water and energy budget imbalances co‐variability depends on the temporal averaging scale, and explain its dependency on how stationary the circulation is in the different model configurations. Finally, we demonstrate the effect of an idealized global warming and convective aggregation on this co‐variability.

## Key Points

- The co‐variability of the atmospheric water and energy imbalances is examined in 3 different model configurations
- We explain the connection of this co‐variability to both the large‐scale circulation and cloud properties
- We demonstrate how this co‐variability can be used to better understand clouds‐circulations coupling

## 1 Introduction

### 1.1 Clouds‐Climate Change Interactions

It is now well established that Earth's climate is changing due to human influence (Masson‐Delmotte et al., 2021). However, large uncertainty still exists regarding the projected impacts and their magnitude in the coming decades. A large portion of this uncertainty can be attributed to clouds, which play an important role in the climate system, mainly by affecting the energy and water budgets. The role of clouds in climate change is manifested by two pathways: (a) the effects of anthropogenic aerosol (tiny, man‐made liquid or solid particles suspended in the air) on clouds (Bellouin et al., 2019; Christensen et al., 2022), which contributes to significant uncertainty in radiative forcing (Bellouin et al., 2019), and (b) the feedbacks which clouds exert on the changing climate (Ceppi et al., 2017; Gettelman & Sherwood, 2016; Nuijens & Siebesma, 2019). Over the last few decades an improved understanding of the processes involved in aerosol‐cloud interactions (ACI) and cloud‐feedbacks has emerged. However, there are still many gaps in our understanding of the interactions between small‐scale processes and the large‐scale climate and circulation (Bony et al., 2015; Schneider et al., 2017; Siebesma et al., 2020).

Both ACI and cloud feedbacks are known to be cloud‐regime dependent (Altaratz et al., 2014; Dagan & Stier, 2020b; Gettelman & Sherwood, 2016; Gryspeerdt & Stier, 2012; Nuijens & Siebesma, 2019), but when studying different cloud regimes, it is also essential to retain a global context. On the one hand, specific cloud regimes are influenced by the large‐scale atmospheric circulation, as large‐scale circulation modulates meteorological (thermodynamic and dynamic) conditions, such as humidity and temperature vertical profiles, large‐scale vertical velocity, etc. However, on the other hand, any anthropogenically driven change in cloud properties may also affect the atmospheric thermodynamic conditions and hence the atmospheric circulation. In addition, changes in one regime could, by driving changes to the water and energy budgets, affect the circulation and other regimes (Dagan & Chemke, 2016). Thus, the cloud scale and the larger climate scale are strongly coupled. In fact, the coupling between clouds and circulation is identified as one of the greatest challenges in climate research (Bony et al., 2015; Siebesma et al., 2020).

Many previous ACI studies (and to a lesser extent also cloud‐feedback studies) adopted a “bottom‐up” approach, that is, study the system from a process‐level perspective and try to quantify individual components of it (Feingold et al., 2016). This approach, while helpful to improve the understanding of the physical processes themselves, falls short in quantifying the total effect on a larger scale, such as the scale of a cloud field, and even more so on the global scale (Feingold et al., 2016). Thus, a “top‐down” approach, which looks for patterns, simple models, and top‐down constraints on the system, is appealing. For example, a top‐down approach was found useful to understand aerosol radiative effect on precipitation (Dagan et al., 2019b, 2021; Hodnebrog et al., 2016; Myhre et al., 2017; Richardson et al., 2018; Samset et al., 2016), and the use of cloud‐controlling factors has been a useful, top‐down approach to study cloud feedbacks (Klein et al., 2017; Myers et al., 2021).

Following the above, we are motivated to define a framework which will enable the investigation of the coupling between cloud properties and the large‐scale circulation in a reduced complexity manner. This framework should capture the interconnections between different cloud regimes and the interconnections between clouds and the atmospheric circulation. We propose such a framework based on the co‐variability of the atmospheric water and energy budget imbalances and demonstrate its usefulness for studying convective aggregation (and specifically the effect of aerosol‐driven diabatic heating on convective aggregation), and the cloud response to an idealized global warming. We examine the co‐variability of the atmospheric water and energy budget imbalances in different model configurations in order to (a) understand its behavior, and (b) understand its connection to cloud properties which helps to establish a better connection between clouds, circulation, and the climate system.

### 1.2 Anthropogenic Perturbations to the Atmospheric Water and Energy Budgets

On monthly and longer timescales (for which the atmospheric storage terms can be neglected), the vertically integrated dry static energy and water budgets are given, respectively, by:

(1) P+Q=div(s)

(2) $$P-E=-\mathrm{div}\left(q_{v}\right)$$

where *P* is the precipitation, *E* is the evaporation, and div(*q*_*v*_) and div(*s*) are the divergence of water vapor (*q*_*v*_) and dry static energy (*s*), respectively (all in units of Wm^−2^). *Q* is the sum of the surface sensible heat flux (*Q*_*SH*_) and the atmospheric radiative heating (*Q*_*R*_) due to radiative longwave (LW) and shortwave (SW) fluxes (F). *Q*_*R*_ can be expressed as the difference between the top of the atmosphere (TOA) and the surface (SFC) fluxes as follows:

(3) $$Q_{R}={(F}_{\text{SW}}^{\text{TOA}}-F_{\text{SW}}^{\text{SFC}})+\left(F_{\text{LW}}^{\text{TOA}}-F_{\text{LW}}^{\text{SFC}}\right)$$

Anthropogenically driven perturbations to the climate system can affect each of the different terms in Equations 1 and 2. For example, an aerosol perturbation could have a local effect on the microphysical cloud processes and hence on the rain production from the clouds (Khain, 2009; Levin & Cotton, 2009), thus directly affecting *P*. In addition, aerosol‐driven changes to the TOA and surface radiative fluxes would perturb *Q*_*R*_ (Dagan et al., 2019b; Myhre et al., 2017), and affect the surface sensible (*Q*_*SH*_) and latent (*E*) heat fluxes. Similar arguments could be presented for an anthropogenic greenhouse gas (GHG) perturbation, which could perturb the rain production (Myhre et al., 2017; Samset et al., 2016), the TOA and surface fluxes and the atmospheric circulation and thus the atmospheric energy and water divergence terms (Muller & O’Gorman, 2011).

On large enough scales (4,000–5,000 km) the water and energy budgets are roughly closed (Dagan et al., 2019a; Dagan & Stier, 2020a; Jakob et al., 2019), and the divergence terms in Equations 1 and 2 are negligible. Consequently, on these scales, any aerosol‐ or GHG‐induced perturbation of, for example, the precipitation, must be balanced by changes in *Q* and *E*. Therefore, improving our understanding of the anthropogenic influence on any one of these terms could enhance our understanding of all of them.

## 2 Methods

We start by examining the co‐variability of the water and energy budget imbalances in three different global model configurations using the same model. The model in use is the ICOsahedral Nonhydrostatic (ICON) Atmospheric GCM (Crueger et al., 2018; Giorgetta et al., 2018; Zängl et al., 2015). ICON, in its GCM mode, uses the ECHAM6 physics packages (Stevens et al., 2013), including a bulk mass‐flux convection scheme (Nordeng, 1994; Tiedtke, 1989) and cloud cover calculated using relative humidity (Sundqvist et al., 1989). The microphysics scheme used here is a modified version of the Lohmann and Roeckner scheme (Lohmann & Roeckner, 1996), which uses prescribed profiles of cloud droplet number concentration over land and ocean (and thus our model does not simulate ACI). The radiative transfer scheme used is the PSrad scheme (Pincus & Stevens, 2013), which implements the solvers from the widely used Rapid Radiation Transfer Model (Mlawer et al., 1997). All simulations were performed at R02B04 horizontal resolution, which corresponds to an approximate grid spacing of 160 km on a rectangular grid, and a terrain‐following vertical sigma‐height grid with 47 levels between the surface and the model top at 83 km.

The three different global model configurations presented here are: radiative‐convective equilibrium (RCE) simulations on an aqua‐planet with no rotation, no diurnal cycle and fixed global sea surface temperature (SST), aqua‐planet with rotation, diurnal cycle and fixed in time meridional SST gradient, thus, without a seasonal cycle, and simulations with land and a seasonal cycle with prescribed SST. The RCE simulations used here were presented in Dingley et al. (2021) and follow the configuration of the RCE model intercomparison project (RCEMIP–[Wing et al., 2018]). In addition to the baseline RCE simulations that were conducted under different SSTs (290 and 305 K), simulations with an added aerosol plume were conducted. The aerosol plume was prescribed using the Max Planck Institute Aerosol Climatology version 2, Simple Plume (MACv2‐SP) model (Stevens et al., 2017). In this study, we present the experiment that was performed with a single plume at latitude = 0°, longitude = 0°, aerosol optical depth of 1.8, and single scattering albedo of 0.8. This produces a strongly absorbing plume, which causes a large heating perturbation. The horizontal shape of the plume is modeled with a Gaussian distribution with aerosol optical depth reducing by a standard deviation every 10° in each direction.

**Figure 1:**
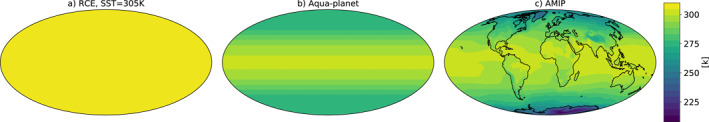
Time‐mean surface temperature in the different ICOsahedral Nonhydrostatic global model simulations: (a) Radiative‐convective equilibrium (RCE), (b) Aqua‐planet, and (c) Atmospheric Model Intercomparison Project (AMIP).

The aqua‐planet simulations with rotation (will be referred to as aqua‐planet simulations hereafter) were presented in Dagan et al. (2019b). The simulations with land follow the Atmospheric Model Intercomparison Project (AMIP) configuration (Gates, 1992) and were presented in Dagan et al. (2021a). In each of the above configurations, the first 4 years of the simulation were used for the analysis, which was chosen as a compromise, that is, a long sampling scale, which still does not average out the signal (at least in the RCE simulations—see Section 6 below for the time sensitivity of the results). For more details about the different model configurations please see relevant papers (Dagan et al., 2019b, 2021a; Dingley et al., 2021). The gradual increase in the level of complexity of the model configuration enables an investigation of the effects of rotation, meridional temperature gradient, land‐sea contrast, and seasonal cycle on the co‐variability of the water and energy imbalance. Figure 1 presents the time‐mean surface temperature in each of the model configurations.

## 3 The Co‐Variability of the Water and Energy Imbalances in Different Model Configurations

**Figure 2:**
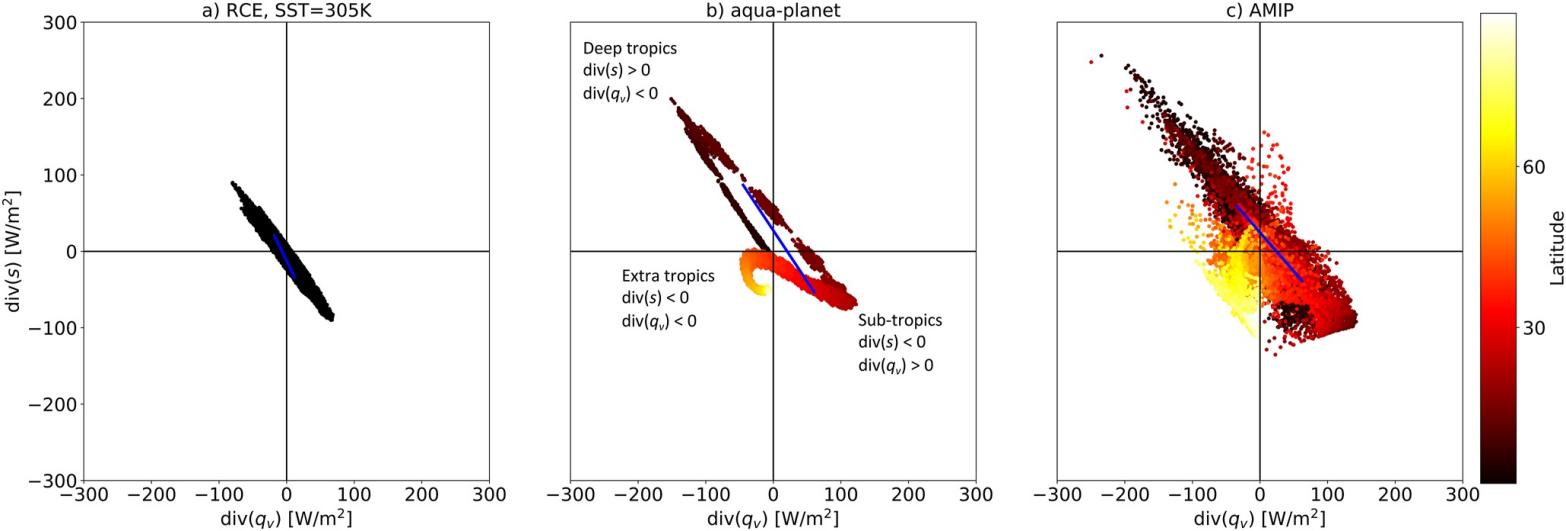
The atmospheric energy [div(*s*)] versus water [div(*q*_*v*_)] imbalance phase‐space—EWIPS for the different ICON global model configurations: (a) Radiative‐convective equilibrium (RCE), (b) Aqua‐planet, and (c) Atmospheric Model Intercomparison Project (AMIP). In (b and c) the global distribution is sorted by the latitude (in absolute value) of each grid point (color‐coded). Each dot represents 4‐year time‐mean of a different model grid point (∼160 km resolution). The blue lines present *d* ‐ the distance between the points on the EWIPS phase‐space that represent the 25th and 75th percentile of the water and energy imbalance (see detail in the text below).

Figure 2 presents the time‐mean co‐variability of the atmospheric water budget imbalance (*P*−*E* or div(*q*_*v*_)) and the atmospheric energy budget imbalance (*P* + *Q* or div(*s*)) for the three model configurations (hereafter referred to as EWIPS ‐ energy vs. water imbalance phase‐space). There is a strong correlation between the atmospheric water and energy imbalances, which depends on the latitude (see Figure 2). In the tropics, the Hadley circulation advects dry‐static energy poleward and water vapor equatorward (Armour et al., 2019). Hence, in the tropics ([−30°,30°]) there is a strong anticorrelation (r = −0.94 for the AMIP simulation) between the residuals of the water and energy budgets—div(*q*_*v*_) and div(*s*), respectively. A simple way to explain this anticorrelation and its link to both atmospheric circulation and clouds (further elaborated in Section 4) can be described as follows—a net water vapor convergence promotes the formation of deep convective clouds and precipitation. Precipitation, in turn, releases latent heat which diverges away efficiently in the tropics (Sobel et al., 2001). The source of the water vapor and the sink of the dry static energy are in subtropical regions, which are dominated by evaporation and shallow clouds. Indeed, this simple picture can explain what happens in the tropical circulations (both the Hadley and Walker circulations), which includes advection of water vapor toward, and dry static energy away from, the equator (or warm SST regions such as the warm pool at the west tropical Pacific) where deep convective clouds are dominant and an opposite trend in the sub‐tropics, where shallow clouds are dominant. In the extratropics, on the other hand, there is net convergence of both water and dry static energy due to advection from lower latitudes (Armour et al., 2019). This correlation between the atmospheric water and energy budgets (arising from the large‐scale circulation) reduces the degrees of freedom of the system and provides a convenient, low‐dimensional phase space for exploring correlations between variables linked to these budgets. Please note that in order to form a spread on the EWIPS in steady‐state (such as seen in Figure 2) a circulation must be present to advect water and energy around the domain. In the absence of a steady‐state circulation, all dots would lie at the origin of the phase‐space as the divergence terms will tend to zero.

We note that there is a “loop” pattern in the aqua‐planet simulation (Figure 2b), which is due to the existence of a double Inter‐Tropical Convergence Zone (ITCZ) in this simulation (cf., Figure 5 below). Due to this double ITCZ pattern, the maximum (minimum) of the divergence of the energy (water) is located north and south of the equator, while just at the equator the values of the divergence terms are close to zero. Thus, starting from a region of strong divergence of water and convergence of energy (the northern hemisphere sub‐tropics—lower right part of the EWIPS), and moving southward, the divergence of energy and convergence of water becomes stronger until a maximum at the northern ITCZ branch which is represented at the upper left part of the EWIPS. Moving further to the south, we encounter a region with a local maximum/minimum in the divergence of water/energy (at the equator—close to the origin of the EWIPS) and back to a region of strong divergence of energy and convergence of water (the southern ITCZ branch—upper left part of the EWIPS again). This trend is manifested as a loop on the EWIPS. In addition, as the aqua‐planet simulation is zonally symmetric, the distribution of the EWIPS is somewhat clustered by latitude bands.

**Figure 3:**
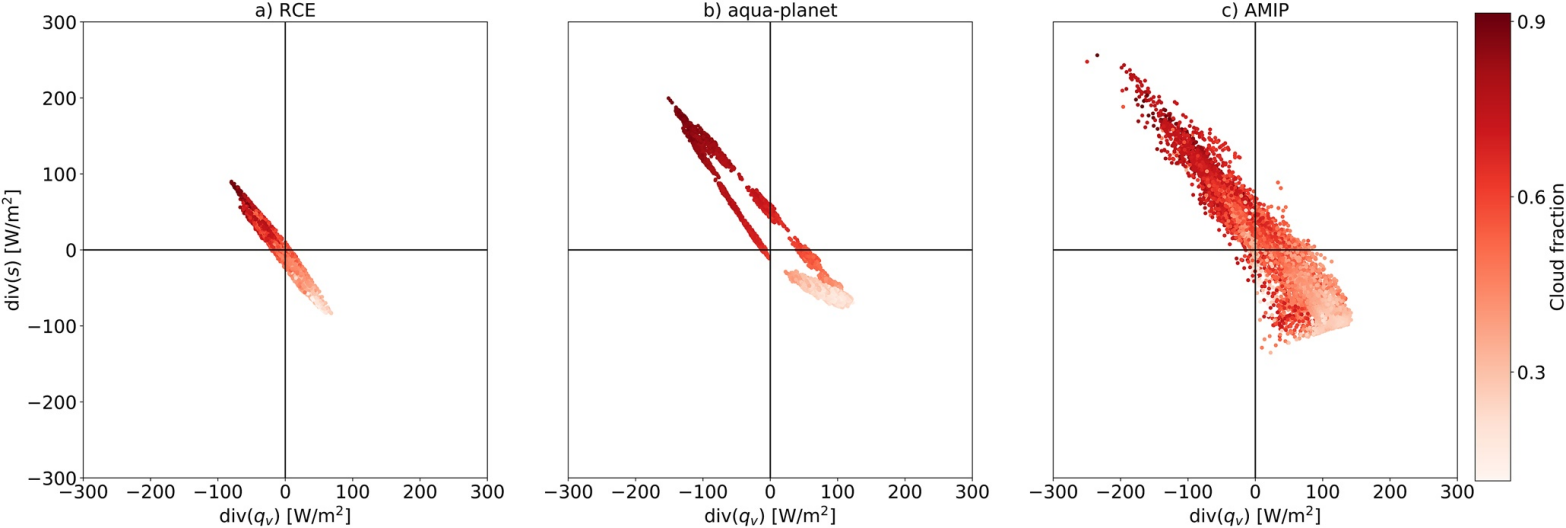
The tropical [−30°,30°] atmospheric energy (div(*s*)) versus water (div(*q*_*v*_)) imbalance phase‐space—EWIPS for the different global model configurations: (a) Radiative‐convective equilibrium (RCE), (b) Aqua‐planet, and (c) Atmospheric Model Intercomparison Project (AMIP). The distributions are sorted by the time‐mean cloud fraction in each grid point (color‐coded).

**Figure 4:**
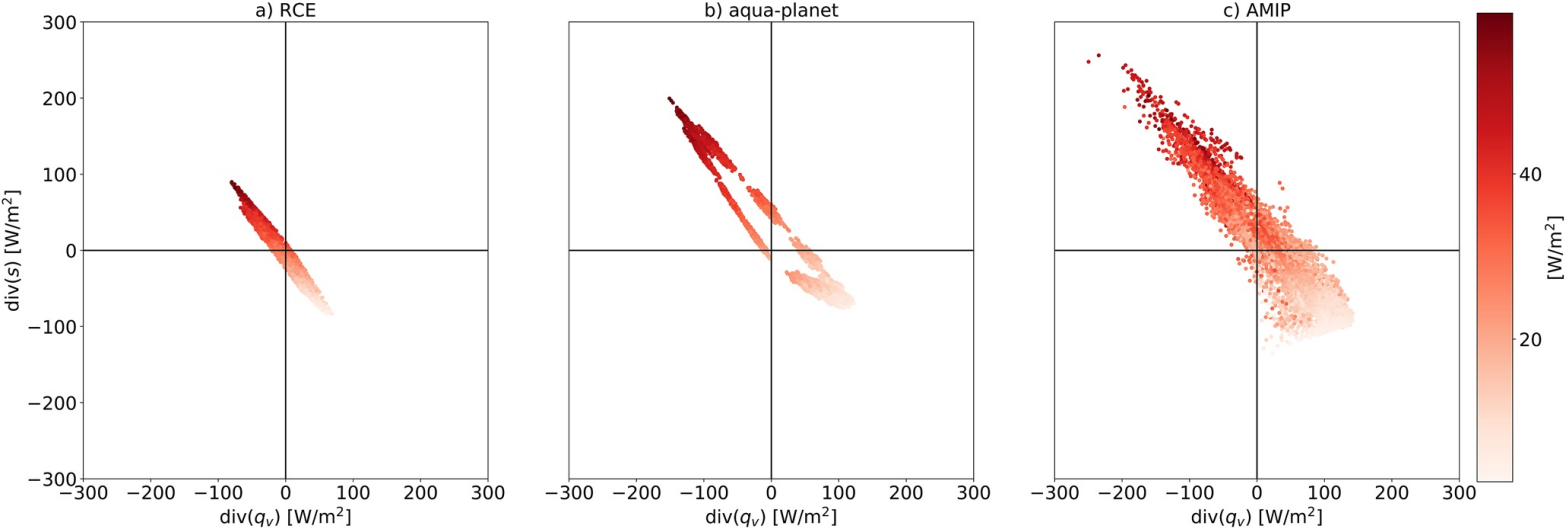
The tropical [−30°,30°] atmospheric energy (div(*s*)) versus water (div(*q*_*v*_)) imbalance phase‐space—EWIPS for the different ICOsahedral Nonhydrostatic global model configurations: (a) Radiative‐convective equilibrium (RCE), (b) Aqua‐planet, and (c) Atmospheric Model Intercomparison Project (AMIP). The distributions are sorted by the time‐mean top of atmosphere (TOA) longwave (LW) cloud radiative effect calculated as the difference between the all‐sky TOA LW flux and the clear sky TOA LW flux.

**Figure 5:**
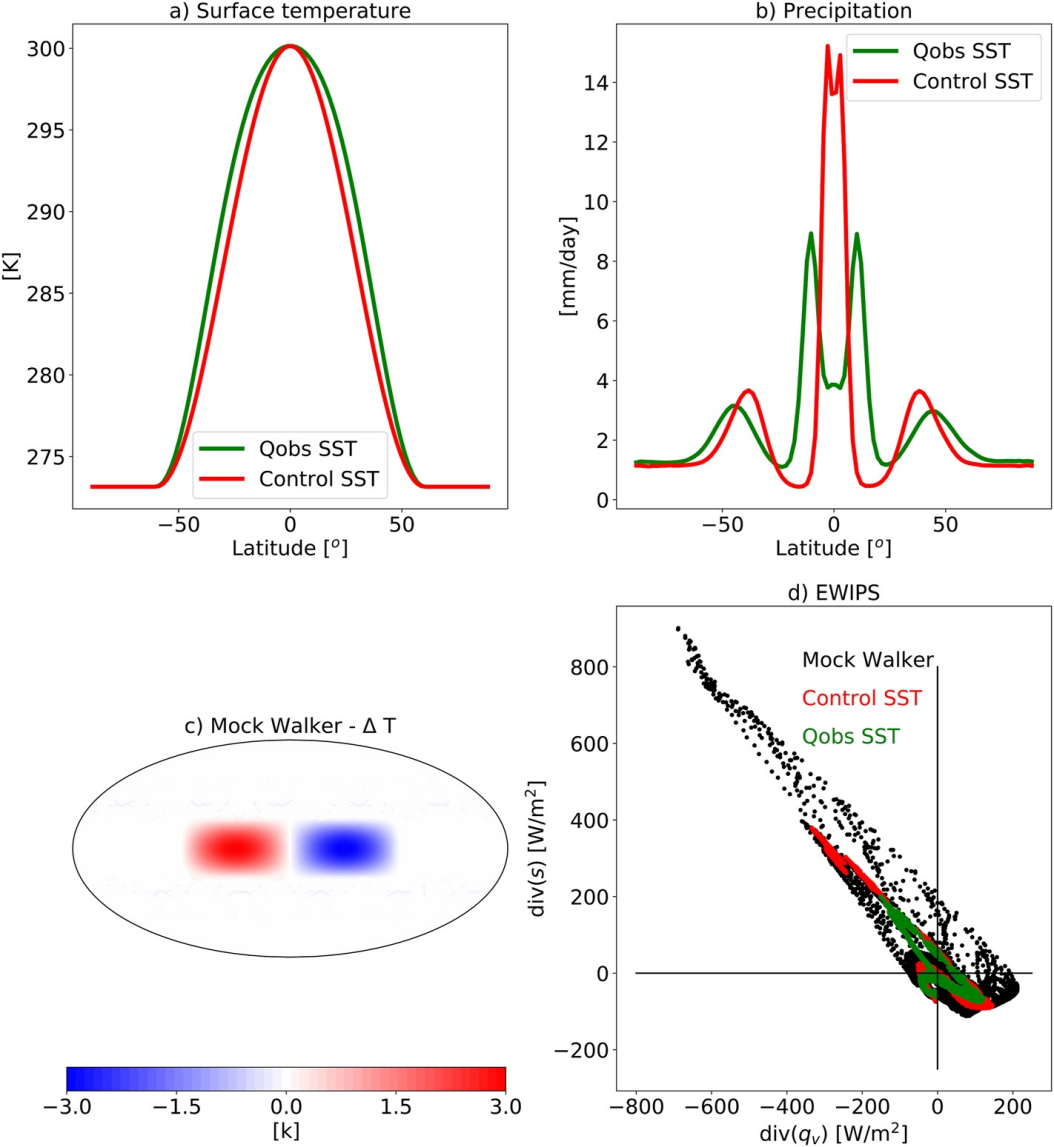
(a) Zonal‐mean surface temperature used to force the aqua‐planet simulations. The Control sea surface temperature (SST) distribution has a steeper meridional SST gradient around the equator compared to Qobs SST distribution (Neale & Hoskins, 2000a, 2000b), (b) Zonal‐ and time‐mean precipitation for the two aqua‐planet simulations with the two different SSTs, (c) The SST perturbation (compared to the Control SST case) in the “mock Walker” simulation, and (d) The atmospheric energy (div(*s*)) versus water (div(*q*_*v*_)) imbalance phase‐space—EWIPS for the different ICOsahedral Nonhydrostatic aqua‐planet simulations. Each dot represents 4‐year time‐mean of a different model grid point (∼160 km resolution).

## 4 Link to Tropical Cloud Properties

The co‐variability of the water and energy budget imbalances is strongly connected to variations in tropical cloud properties (see Figures 3 and 4). In the deep tropics, where convergence of water and divergence of energy are dominant, the average cloud fraction is higher (Figure 3) and the TOA LW cloud radiative effect (Figure 4) is larger compared to the sub‐tropics (in which divergence of water and convergence of energy are dominant). The atmospheric water and energy budgets are strongly coupled to both the large‐scale circulation of the atmosphere (Armour et al., 2019; Dagan & Stier, 2020a) and to cloud properties (Figures 3 and 4), hence providing a link between them.

Due to the strong coupling between clouds and circulation (Bony et al., 2015; Siebesma et al., 2020), any anthropogenic change to regional cloud properties would affect the advection of water and energy (Dagan & Chemke, 2016), the circulation, and hence is expected to affect the EWIPS phase‐space. The same is true from the other direction; any anthropogenic circulation changes would be manifested as changes in the water and energy advection, and hence in the EWIPS phase‐space, and would be accompanied by changes to the cloud properties (Voigt et al., 2021). The EWIPS perspective provides a framework that enables the investigation of the coupling between cloud and circulation in a reduced complexity manner.

In addition, examining variations in the EWIPS under different environmental conditions enables investigation of the interactions between the different cloud regimes (which occupy different parts of the phase‐space—Figure 2) and the effects of each regime on the general circulation. Changes in one cloud regime could, by driving changes to the water and energy divergence terms, affect the circulation and the other regimes as well (Dagan & Chemke, 2016).

Moreover, we note that convective aggregation, which could play a significant role in the climate system (Wing, 2019), is also expected to generate an anticorrelation between div(*s*) and div(*q*_*v*_), as the circulation associated with convective aggregation advects water vapor toward, and energy away from, the center of the aggregation. Hence, in the RCE configuration [which generates convective self‐aggregation (Dingley et al., 2021)] we see a similar anticorrelation as in the tropics of the aqua‐planet and AMIP simulations even without large‐scale circulation such as the Hadley circulation. Thus, the co‐variability of the water and energy budget imbalances could also be used to study convective aggregation (as will be elaborated upon in Section 7).

## 5 Zonal and Meridional Tropical Circulations Effects on the EWIPS

The wider distribution on the EWIPS in the AMIP simulation compared to the aqua‐planet simulation (Figure 2) is caused by the land‐sea contrast that breaks the zonal symmetry and introduces zonal circulation (such as the Walker circulation). The zonal circulation generates preferential locations for deep convection (such as the warm pool at the west Pacific) and widens the possible conditions on this phase‐space for a given latitude. The preferential locations for deep convection are supported by stronger dry static energy divergence and water vapor convergence compared to a zonally symmetric world, manifested as points further up the upper‐left side of the EWIPS. In addition, a strengthening of the meridional circulation (the Hadley circulation) could also affect the spread on the EWIPS by strengthening the water vapor and energy divergence. To examine these effects, we have conducted additional aqua‐planet simulations with different prescribed SSTs. As a first step, we force the model with a steeper SST meridional gradient around the equator in a zonally symmetric aqua‐planet. Steeper SST meridional gradients are expected to generate a stronger Hadley circulation and a narrower ITCZ (and even generate a single ITCZ instead of a double ITCZ) (Neale & Hoskins, 2000a, 2000b). Figure 5 presents the zonal mean precipitation (Figure 5b) for the two different aqua‐planet simulations with different zonal mean SST profile (Figure 5a). Indeed, the SST profile with the steeper gradient around the equator [Control SST compared to Qobs SST (Neale & Hoskins, 2000a, 2000b)], generates a narrower and stronger ITCZ, as expected (Neale & Hoskins, 2000a, 2000b). In addition, in the Qobs SST case the precipitation demonstrates a clear double ITCZ pattern, which is much less significant in the case of the Control SST case. This stronger and narrower ITCZ requires a stronger divergence of dry static energy and convergence of water vapor. Hence, the spread on the EWIPS is larger (Figure 5d ‐ please also see the discussion about the effect of convective aggregation on the EWIPS in Section 7). In addition, as was mentioned above, we note that the double ITCZ pattern seen in the Qobs SST case produces a “loop” in the EWIPS with a lower magnitude of convergence of water vapor and divergence of dry static energy just at the equator compared with at the ITCZ (which is off the equator to the north and south—see this “loop” in Figure 2). This “loop” pattern is not seen in the Control SST case with the single ITCZ (Figure 5d).

To isolate the role of zonal circulation on the EWIPS (noting that the difference between the aqua‐planet and AMIP simulations is caused by both the addition of zonal circulation and seasonal cycle) we introduce an aqua‐planet simulation with a “mock‐Walker” circulation without a seasonal cycle. The “mock‐Walker” simulation is done by adding a wavenumber‐2 SST dipole on top of the Control SST distribution (δSST), which spans a half‐hemisphere and takes the form:

(4) $$\delta \mathrm{SST}=3\cos(2\lambda )\cos\left(\frac{\pi }{2}\left[\frac{\phi }{\phi_{0}}\right]\right)\;\;\;\;\mathrm{for:}\;\frac{\pi }{4}<\lambda <\frac{3\pi }{4}$$

with *λ* the longitude, ϕ the latitude, and $\phi_{0}=\frac{\pi }{6}=30^{\circ }$ is the latitudinal width of the perturbation. The amplitude of the dipole is ±3 K (Figure 5c). These simulations were presented in Williams et al. (2021). Introducing a zonally asymmetric SST pattern generates a zonal circulation with preferential locations for deep convective formation (the warm pool), thus significantly widens the spread on the EWIPS (Figure 5d). As expected, the points on the far upper‐left side of the phase‐space are associated with the warm pool.

## 6 The Time‐Dependence Variability of the EWIPS

**Figure 6:**
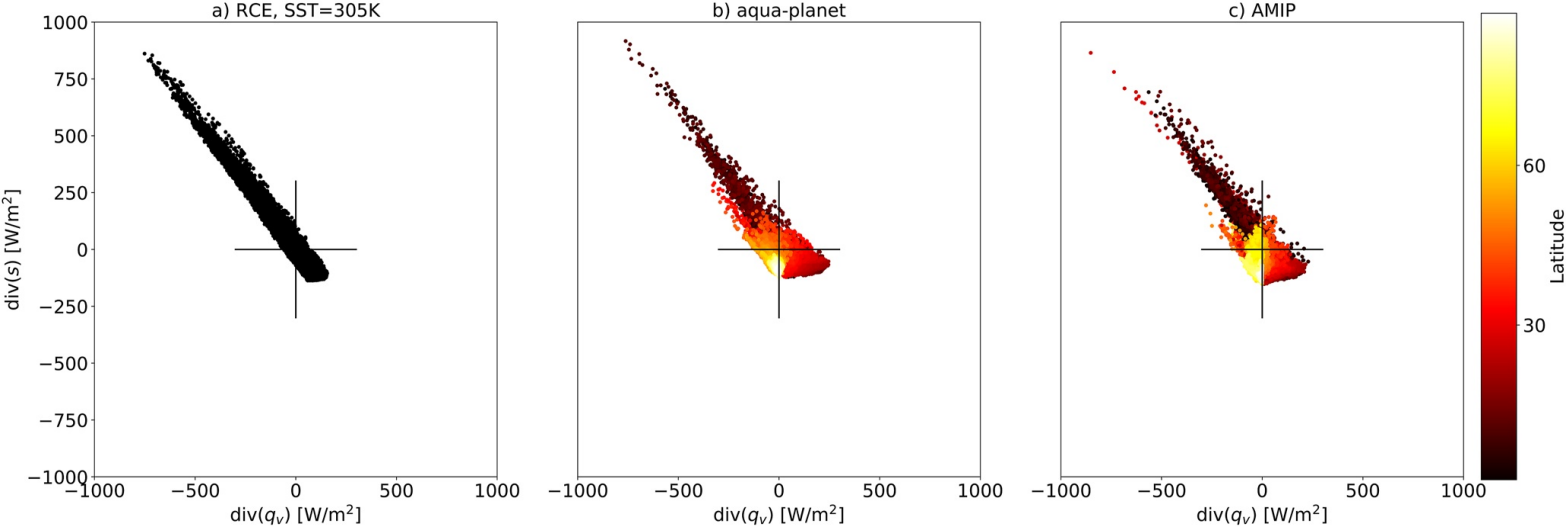
The atmospheric energy (div(*s*)) versus water (div(*q*_*v*_)) imbalance phase‐space—EWIPS for the different global model configurations: (a) Radiative‐convective equilibrium (RCE), (b) Aqua‐planet, and (c) Atmospheric Model Intercomparison Project (AMIP). In (b and c) the global distribution is sorted by the latitude (in absolute value) of each grid point (color‐coded). Each dot represents 1 month time‐mean of a different model grid point (∼160 km resolution). This figure is similar to Figure 2 but for shorter time average. The black lines demonstrate the spread presented in Figure 2 for comparison.

The results above are based on long (4‐year) averages of the water and energy imbalances. The spread on the EWIPS is expected to be dependent on the averaging time (the variability should generally decrease with longer averaging times until a certain point). For example, in Figure 6 we present the EWIPS for the 3 different model configurations based on a single month averaging. It demonstrates a much larger spread compared to the 4‐year averages (please note the black crosses in Figure 6 demonstrating the spread presented in Figure 2). To examine and quantify this time‐dependence in the variability, we define a distance *d* on the EWIPS phase‐space. *d* is calculated as the distance [in units of W/m^2^] between the points on the EWIPS phase‐space that represent the 25th and 75th percentile of the water and energy imbalance (see examples in Figure 2), hence, captures the variability on the phase‐space.

**Figure 7:**
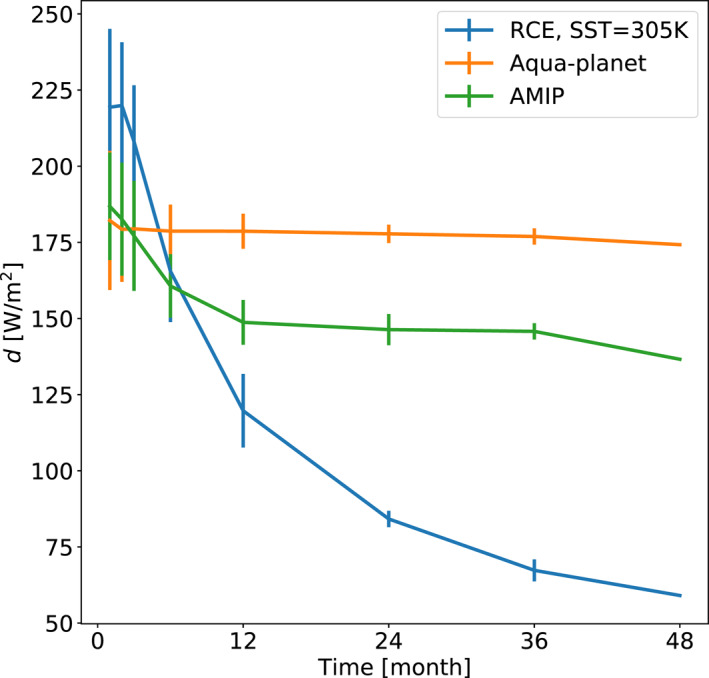
A distance *d* on the EWIPS phase‐space versus the time the EWIPS was averaged over for the three different global model configurations. *d* is calculated as the distance [in units of W/m^2^] between the points on the EWIPS phase‐space that represent the 25th and 75th percentile of the water and energy imbalance in the tropics [−30°,30°] (see examples in Figure 2). For each time *d* was calculated based on all the potential time‐windows with that length in the 4‐year long simulation. The vertical lines represent the standard deviation of all these time‐windows.

Figure 7 presents *d* as a function of the averaging time for the three model configurations. It demonstrates that *d* generally decreases with averaging time as expected. However, the behavior is different between the different model configurations, which provides information on how stationary the circulation is. In the RCE configuration, where the domain is homogenized in terms of SST and no rotation is applied, there are no preferential locations for the deep convection formation. Hence, the clusters of convection are randomly distributed for long enough times. This homogenization results in narrower distribution on the EWIPS as the averaging time increases—hence *d* decreases with time. In contrast, in the aqua‐planet simulation, the imposed meridional SST gradient and the rotation of Earth generate a preferential location of the deep convective formation (the ITCZ). These deep convective clouds are associated with water vapor convergence and dry static energy divergence (due to the latent heat release by precipitation), and a more stationary circulation. In addition, in the absence of seasonal cycle (the case in the aqua‐planet simulation), the ITCZ's location hardly changes with time. Hence, in the aqua‐planet simulation *d* does not vary much with time. In the AMIP simulation, the meridional and zonal temperature gradients also generate preferential deep convective formation locations and more stationary large‐scale circulation compared with the RCE. However, the seasonal cycle shifts these locations with time on a sub‐yearly timescale (i.e., the ITCZ location changes with the seasons). Hence, in the AMIP simulation *d* decreases with time up to a year‐long average and stay roughly constant for longer timescales.

## 7 Connection With Convective Aggregation and SST Dependency

Convective aggregation is known to affect the variance of moist‐static energy (Wing, 2019). As was mentioned above, deep‐convection aggregation or clustering is associated with convergence of water vapor toward the cluster and divergence of dry static energy away of the cluster, hence, it is associated with a wide spread on the EWIPS. To demonstrate it we use the RCE simulations presented in Dingley et al. (2021) under different SSTs. In Dingley et al. (2021) it was shown that, in ICON RCE simulations, the level of aggregation of the convection is higher under higher SST [305 K compared to 290 K, in agreement with some previous studies (Emanuel et al., 2014; Wing & Emanuel, 2014)] and that homogenizing the LW radiation prevents the convection from aggregating (Dingley et al., 2021) as the LW feedback was shown to be a necessary process (Coppin & Bony, 2015; Muller & Held, 2012).

**Figure 8:**
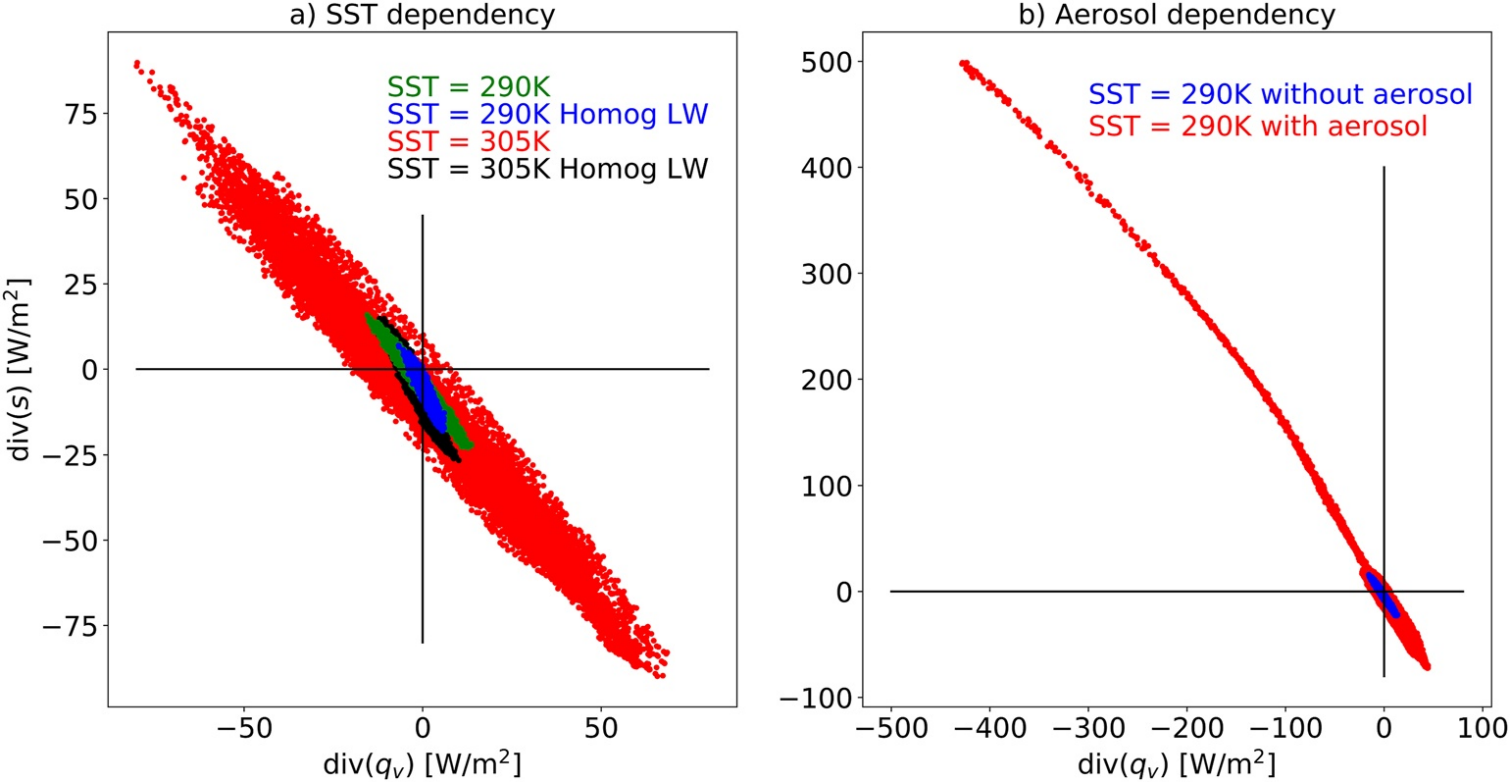
The atmospheric energy (div(*s*)) versus water (div(*q*_*v*_)) imbalance phase‐space—EWIPS for the different Radiative‐convective equilibrium (RCE) simulations. In (a) 4 different RCE simulations are presented with two different sea surface temperatures (SSTs) (290 and 305 K) and with/without homogenization of the longwave radiation (Homog LW) for each SST. In (b) two simulations are presented with the same SST (290 K) but with and without a plume of absorbing aerosols which force the convection to aggregate. Each dot represents 4‐year time‐mean of a different model grid point (∼160 km resolution).

Figure 8a presents the EWIPS for the RCE simulations. It demonstrates that the spread on the phase‐space increases with the SST as the convection becomes more aggregated. We note that even for a given circulation or level of aggregation the spread on the EWIPS should increase with SST as the air contains higher water vapor concentrations (for a given relative humidity) and higher dry static energy. However, for a given SST, homogenizing the LW radiation reduces the spread on the EWIPS, specifically for the higher SST case (Figure 8a), which was shown to have a higher level of convective aggregation (Dingley et al., 2021).

In addition, it was shown that convection can be forced to aggregate by local diabatic heating caused by absorbing aerosols (Dingley et al., 2021). The local aerosol diabatic heating generates a large‐scale thermally driven circulation (Dagan et al., 2019b). This circulation supports the convective aggregation by converging water vapor and diverging dry static energy, hence, leading to a much wider spread on the EWIPS compared to a simulation without an aerosol plume (Figure 8b). Moreover, when the convection clusters around the stationary aerosol perturbation the circulation is much more stationary in time, hence the spread on the EWIPS is wider (Figure 8b).

**Figure 9:**
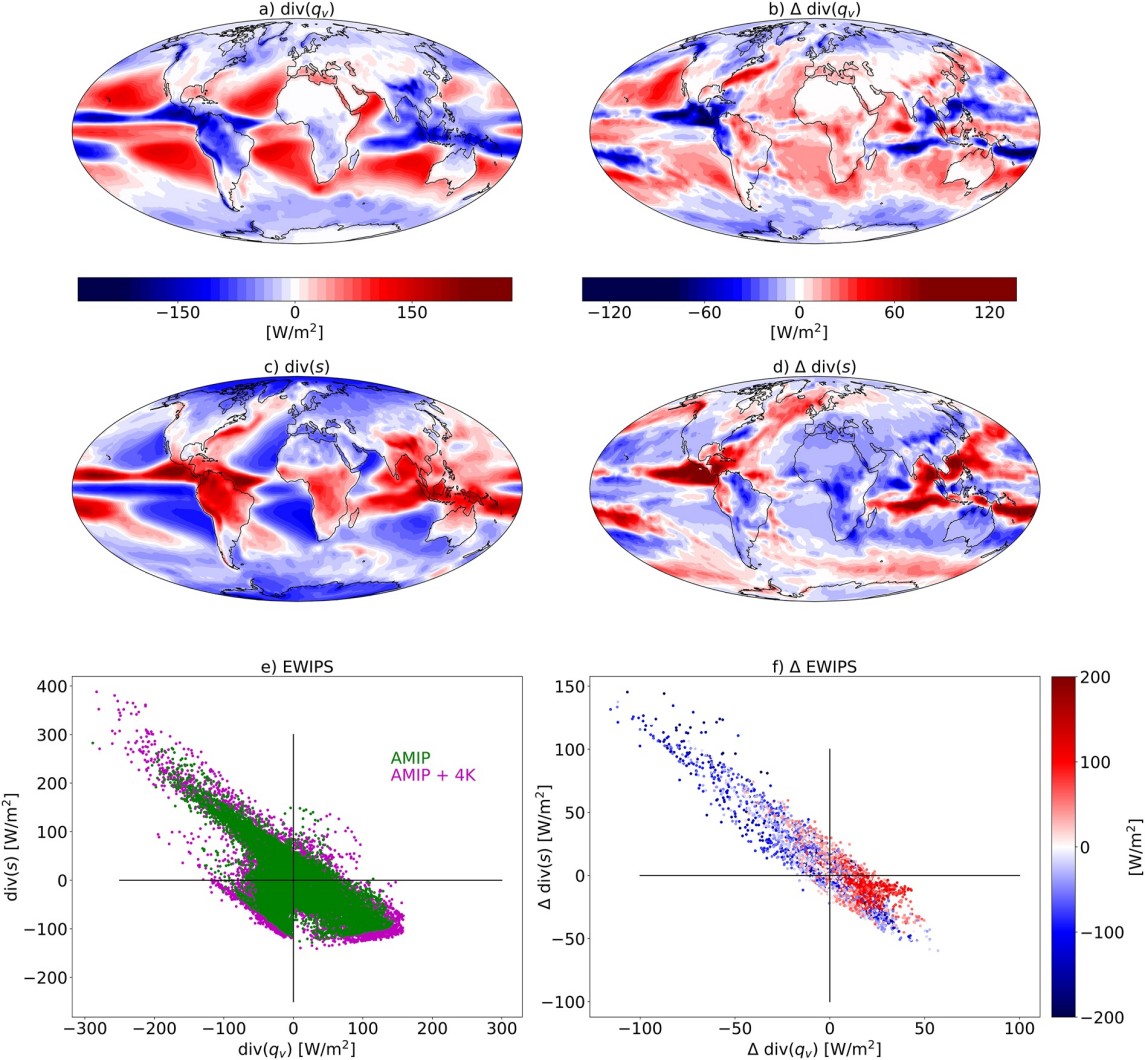
Maps of the time‐mean: (a) water vapor divergence (div(*q*_*v*_)) in the baseline Atmospheric Model Intercomparison Project (AMIP) simulation, (b) change in the water vapor divergence under a +4K uniform warming (AMIP+4K‐AMIP), (c) dry static energy divergence (div(*q*_*v*_)) in the baseline AMIP simulation, and, (d) change in the dry static energy divergence under a +4K uniform warming (AMIP+4K‐AMIP). (e) The atmospheric energy (div(*s*)) versus water (div(*q*_*v*_)) imbalance phase‐space—EWIPS for the AMIP and AMIP+4K simulations and (f) the change in the atmospheric energy imbalance versus the change in the water imbalance under a +4K uniform warming (AMIP+4K‐AMIP) over the tropics [−30°,30°]. The color coding in (f) presents the water vapor divergence (div(*q*_*v*_)) in the baseline AMIP simulation.

Next, we demonstrate how the EWIPS perspective can be used for understanding the coupling of clouds and circulation under an idealized global warming scenario in AMIP simulation (a uniform SST increase of 4K). Figure 9 presents the time‐mean divergence of (a) water vapor and (c) dry static energy in the baseline AMIP simulation. These maps clearly demonstrate the spatial anticorrelation between the water and energy budget imbalances in the tropics. Specifically, it shows the convergence of water and divergence of dry static energy at the ITCZ and the opposite in the sub‐tropics. The net convergence of both water and energy at mid to high latitudes is also evident. Figures 9b and 9d present the change in the water and energy divergence terms under a uniform +4 K SST AMIP experiment. Over most of the tropical oceans there is a general trend of strengthening of the baseline conditions; that is, regions that were dominated by divergence of water and convergence of energy (such as the sub‐tropics) demonstrate stronger water vapor divergence and energy convergence under warmer conditions. The exact opposite trend is seen over the deep tropical oceans—stronger water vapor convergence and energy divergence under warmer conditions. This trend resembles the “wet‐get‐wetter‐dry‐get‐dryer” paradigm (Held & Soden, 2006). This “wet‐get‐wetter‐dry‐get‐dryer” trend is manifested as a wider spread on the EWIPS under warmer conditions (Figure 9e). Examining the change in the water and energy budget imbalances in each grid point in the tropics on the EWIPS (Figure 9f) demonstrates that the anticorrelation between the water and energy imbalances is present also when examining the changes under global warming. Furthermore, it demonstrates that the change in the location on the EWIPS depends on the initial location, which is another manifestation of the “wet‐get‐wetter‐dry‐get‐dryer” trend, that is, generally, grid points that were dominated by divergence of water and convergence of energy in the baseline conditions (at the lower‐right part of the EWIPS), experience stronger divergence of water and convergence of energy under warmer conditions (at the lower‐right part of Figure 9f).

**Figure 10:**
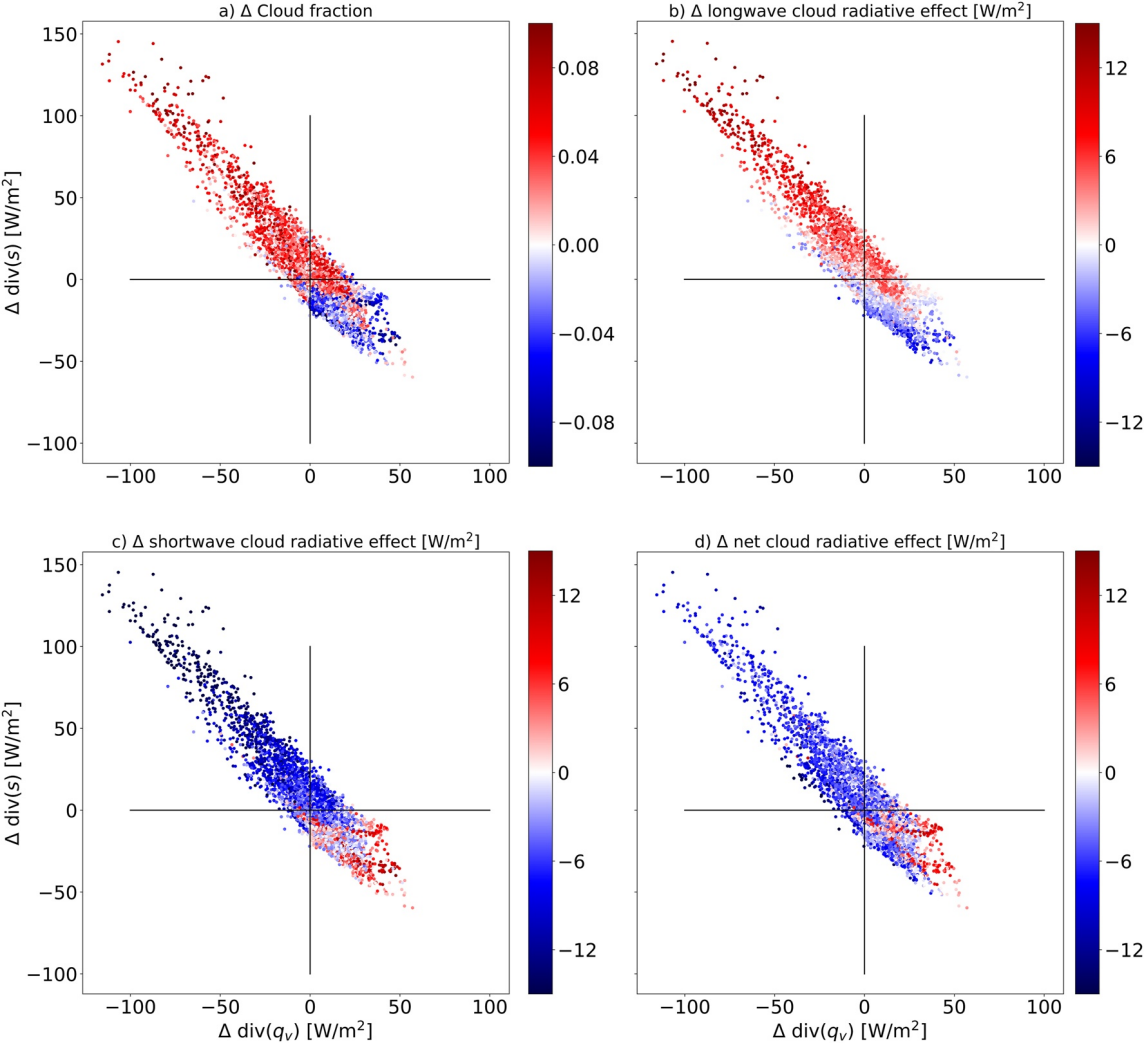
The tropical [−30°,30°] change in the atmospheric energy versus the change in the water imbalance under a +4K uniform sea surface temperature (SST) warming (AMIP+4K‐AMIP). The color coding presents: (a) the change in cloud fraction, (b) the change in top of atmosphere (TOA) longwave (LW) cloud radiative effect, (c) the change in TOA shortwave (SW) cloud radiative effect, and (d) the change in the net TOA cloud radiative effect (SW+LW) under a +4K uniform SST warming (AMIP+4K‐AMIP).

Finally, we seek to answer the question: how does this idealized global warming affect regional cloud properties and how are these changes linked to changes in the atmospheric circulation? The EWIPS framework can nicely answer this question. Figure 10 presents the change, over the tropics [−30°,30°], in the water and energy imbalances under the uniform SST increase simulation (similar to Figure 9f) with a color coding sorting the different grid points by the change in cloud properties. It demonstrates that geographical locations experiencing stronger water vapor convergence and dry static energy divergence under global warming (upper‐left part of the phase space) are associated with an increase in cloud cover and TOA LW cloud radiative effect and a decrease in TOA SW cloud radiative effect. Meanwhile, geographical locations experiencing stronger water vapor divergence and dry static energy convergence under global warming (lower‐right part of the phase space) are associated with the opposite trend ‐ a decrease in cloud cover and TOA LW cloud radiative effect and an increase in TOA SW cloud radiative effect. These results demonstrate that an increase in water vapor convergence under warmer conditions will drive an increase in the deep convective and anvil cloud coverage (hence the increase in the TOA LW cloud radiative warming and the SW cooling), which will also be associated with an increase in precipitation and latent heat release, thus also with an increase in dry static energy divergence.

Figure 10 clearly demonstrates the strong coupling between changes in the atmospheric water budget, in the atmospheric energy budget, and in cloud properties in the tropics under climate change. It demonstrates how using the EWIPS perspective helps connect clouds and circulation in a reduced complexity manner. For example, and keeping in mind that the system is highly coupled, one can estimate cloud properties changes from circulation (or specifically water vapor and dry static energy divergence) changes and vice versa. In addition, it shows that due to the strong anticorrelation between the water and energy budgets imbalances in the tropics, a change in the water/energy imbalance is associated with the opposite change in the energy/water imbalance.

## 8 Summary

By strongly affecting the radiation budget and by releasing latent heat, clouds affect the large‐scale atmospheric circulation. Concomitantly, clouds are affected by the meteorological conditions such as large‐scale vertical velocity and humidity and temperature vertical profiles, which are modulated by the large‐scale circulation. Thus, there exists a strong, two‐way, coupling between clouds and the large‐scale atmospheric circulation. While this two‐way coupling is well appreciated, the research around the role of clouds in the climate system suffers from our limited ability to connect the cloud scale and the larger global scale in our research tools.

In this study, we examine the co‐variability of the atmospheric water and energy budget imbalances in three different global model configurations ‐ RCE, aqua‐planet and simulations with prescribed SSTs and land (AMIP). We explore the differences between the different model configurations and examine the effect of rotation, meridional, and zonal temperature gradients and seasonal cycle on this co‐variability. It is shown that the atmospheric water and energy budget imbalances demonstrate a strong anticorrelation in the tropics and subtropics with a strong water vapor convergence and dry static energy divergence in the deep tropics and vice versa in the subtropics. This strong anticorrelation is inherently linked to both the tropical circulation and clouds. We propose that this strong anticorrelation between the atmospheric water and energy budget imbalances and its link to cloud properties in the tropics could be used as a simplified framework to study the coupling between clouds and circulation. For example, if the time‐mean water vapor imbalance can be measured in the tropics (e.g., by surface precipitation and evaporation measurements), one can estimate the dry static energy divergence (Figure 2) and the cloud properties (Figures 3 and 4) in this region. Please note that, while the physical explanations mentioned above are known, we believe that presenting and examining the cloud‐circulation coupling using the EWIPS perspective helps to reduce the complexity of the system and hence improve our understanding. In addition, we anticipate that shifting our perspective to a framework that directly accounts for the conservation of water and energy, and for the interconnections between different cloud regimes, forms a more holistic understanding of the cloud‐circulation coupled system.

By using idealized simulations with variations in the SST, we have demonstrated the effects of zonal and meridional circulations on the EWIPS. We have shown that a stronger meridional circulation (forced by a stronger meridional SST gradient around the equator) generates a larger spread on the EWIPS due to stronger water vapor convergence and dry static energy divergence in the deep tropics and vice versa in the sub‐tropics. A similar trend is seen when convective aggregation is generated (either spontaneously under higher SSTs or forced by aerosol diabatic heating) in the RCE simulations. The effect of a zonal circulation on the EWIPS is examined using an idealized mock‐Walker circulation in the aqua‐planet configuration and is also shown to increase the spread on the EWIPS due to the formation of preferential locations for deep convective development.

In addition, we have examined the time‐dependency of the EWIPS framework and demonstrated, using the different model configurations, that this time‐dependency could be used to explore how stationary the circulation is. That is to say that a more stationary circulation is manifested as a weaker time‐dependency of the spread on the EWIPS, while a more random circulation associated with deep convective clustering, as seen in the RCE simulations, is manifested as a faster decrease in the spread on the EWIPS with time.

Finally, we demonstrated a possible use of the water and energy imbalance framework to the study of clouds‐circulation coupling under an idealized global warming. We demonstrated that a “wet‐get‐wetter‐dry‐get‐dryer” type of response under global warming (Held & Soden, 2006) manifests as an increase in the spread on the EWIPS. Geographical locations that are dominated by divergence of water and convergence of energy in the baseline simulation (such as the sub‐tropics) demonstrate stronger water vapor divergence and energy convergence under warmer conditions. The exact opposite trend is seen over the deep tropical oceans—stronger water vapor convergence and energy divergence under warmer conditions, hence the spread on the EWIPS increases. We have further demonstrated how these changes in the EWIPS are related to changes in the cloud properties. We have shown that geographical locations experiencing stronger dry static energy divergence and water vapor convergence under warmer conditions are associated with an increase in cloud cover and TOA LW cloud radiative effect and with a decrease in the TOA SW cloud radiative effect. The opposite cloud properties response is associated with geographical locations experiencing stronger energy convergence and water vapor divergence under warmer conditions. Thus, we suggest that using the EWIPS perspective could help us better understand and connect clouds and circulation changes under climate change.

In the literature, one can find many frameworks that use different spaces to study clouds and their response to anthropogenic perturbations. These include spaces based on mid‐tropospheric vertical velocity (Bony et al., 2004), the cloud center of gravity versus water mass (Heiblum et al., 2016), the cloud droplet concentration versus liquid water path (Glassmeier et al., 2021; Hoffmann et al., 2020), etc. The added value of the EWIPS on top of the previous frameworks is the direct connection to top‐down constraints on the system, that is, the water and energy budgets. Recent work demonstrated the importance of considering water conservation in cloud resolving simulations, demonstrating that the representation of the water vapor convergence into the domain can determine the cloud response to aerosol perturbations (Dagan et al., 2022). Thus, exploring the sensitivity of the water and energy divergence to anthropogenic perturbations and its connection to cloud properties changes is of great importance.

The rise of global storm resolving models (Stevens et al., 2019) implicitly addresses the interactions between the local cloud properties and the large‐scale circulation. However, the large volume of data these models produced requires, again, the development of simplified and reduced complexity frameworks to examine the interaction between clouds and circulation. The EWIPS perspective could become a useful tool in large domains, or even global, cloud resolving simulations analysis by providing a simple, low‐dimensional representation of clouds and circulation across different regimes. Finally, we note that this study is based on a single model and that the results could, to some degree, be model‐dependent (e.g., due to the use of a different convection scheme). Comparing the spread on the EWIPS and its connection to cloud properties in different models could be a simple and informative way to examine inter‐model differences.

## Conflict of Interest

The authors declare no conflicts of interest relevant to this study.

## Data Availability

The data from the aqua‐planet simulations can be found online at: https://doi.org/10.5281/zenodo.3242319 (Dagan et al., 2019c). The data from the AMIP simulations can be found online at: https://doi.org/10.5281/zenodo.4448345 (Dagan et al., 2021b). The RCE simulations are freely available in NetCDF format online at http://dx.doi.org/10.5285/1a86e0326e1346febf121eca83bf1f08 (Dingley et al., 2021b).
